# Thermal and Structural Characterization of Geopolymer-Coated Polyurethane Foam—Phase Change Material Capsules/Geopolymer Concrete Composites

**DOI:** 10.3390/ma12050796

**Published:** 2019-03-07

**Authors:** Ahmed Hassan, Yasir Rashid, Abdel-Hamid I. Mourad, Najif Ismail, Mohammad Shakeel Laghari

**Affiliations:** 1 College of Engineering, United Arab Emirates University, P.O. Box 15551, Al Ain, United Arab Emirates; ahmed.hassan@uaeu.ac.ae (A.H.); yasir.rashid@uaeu.ac.ae (Y.R.); mslaghari@uaeu.ac.ae (M.S.L.); 2 School of Engineering, Wellington Institute of Technology, Private Bag 39803, Lower Hutt 5045, New Zealand; najif.ismail@weltec.ac.nz

**Keywords:** phase change material, geopolymer concrete, polyurethane foam, thermal performance, thermal insulation, compressive strength

## Abstract

The thermal and structural performance of geopolymer-coated polyurethane foam–phase change material capsules/geopolymer concrete composites was investigated. Three groups of concrete composites were prepared. The first was pure geopolymer (GP, control sample), the second was a GP/polyurethane foam (F) concrete composite, and the third was GP-coated polyurethane foam-phase change material capsules (GP-F-PCM)/GP concrete composites. Three different percentages of foam and GP-F-PCM capsules (25%, 50%, and 75%) were used in the composites. Thermal and U-value tests were conducted for all composites to characterize their peak temperature damping and insulation performances. The addition of 75% foam has been noticed to increase the back-surface temperature by 5.9 °C compared to the control sample. This may be attributed to the degradation of foam into low molecular constituents in the presence of a strong alkali. However, a temperature drop of 12.5 °C was achieved by incorporating 75% of GP-F-PCM capsules. The addition of 50% foam as a sandwich layer between two halves of a geopolymer concrete cube is also investigated. It was found that inserting a foam layer reduced the back-surface temperature by 3.3 °C, which is still less than the reduction in the case of GP-F-PCM capsules. The compressive strength was tested to check the integrity of the prepared concrete. At 28 days of aging, the compressive strength dropped from 65.2 MPa to 9.9 MPa with the addition of 75% GP-F-PCM capsules, which is still acceptable for certain building elements (e.g., nonloadbearing exterior walls). Generally, the best results were for the GP-F-PCM composite capsules as a heat insulator.

## 1. Introduction

Rapid economic growth and a rise in living standards have increased the energy demand at a staggering rate, with an associated surge in climate change [1]. World energy consumption was 4331 million tons in 2015, which rose by an average of 1.5% per year from 2010 to 2015. The report revealed that the consumption of coal and gas increased at a respective rate of 1.7% and 1.1% annually in the same period, posing several sustainability and environmental challenges [2]. Space heating and cooling account from 18% to 73% of total building energy. This variation in the share of energy consumption in residential and commercial buildings is due to climatic conditions, the development status of the countries, the economic status of the people, and the energy policies implemented [3]. This challenge has prompted different renewable energy strategies in the building components of roofs [4], walls [5], and underfloor heating [6] to minimize the indoor energy demand by protecting it from the harsh outdoor environment. Apparently, increasing the heat capacity and storage capability of building envelopes is a key approach for reducing the fluctuation of indoor temperatures [7]. Phase change materials (PCMs) can absorb and release a lot of latent heat during their phase transitions in a narrow temperature range [8]. Generally, there are three main methods commonly used to design the latent thermal energy storage (LTES) components [9]. The first is to encapsulate PCM into wall boards. Aiming at improving the efficiency, different design geometries such as PCM wall boards coupled with vacuum isolation panels [10,11] and placement of a PCM composite wall plate within a multiple-layered building envelope [12,13,14] were proposed recently. The second method is to use PCM brick. A hollow thermal-insulation brick, including clay bricks [15], glass bricks [16,17], and gypsum bricks [18] that have holes inside, is suitable to contain PCMs for indoor temperature control. The third method is to produce PCM concrete, which is considered the most realistic approach to developing monolithic structures.

A study compared the thermal performance of Micronal (BASF, Ludwigshafen, Germany) panel with the panel containing PCM and observed a temperature difference of 4 °C at the maximum. The research experienced a leakage problem of PCM up to 67% [19]. The leakage problem was solved by using a hydrophobic nano powder that absorbed exuded PCM and did not allow the PCM to interact with water during mixing [20]. If PCM interacts with water, it disturbs the movement of the water molecules necessary for the hydration of cement [8] and also the formation of possible phase interfaces by the wax [21], causing a reduction in the compressive strength of concrete. Later investigation exploited the use of hydrophobic coated expanded perlite (EP) as an effective containment of PCM and tested its effect on leakage and thermal performance. Hydrophobic coated EP performed better in leak-proofing and the integration of PCM composites increased the thermal inertia of the panel more than double as compared to the reference [22]. The effect of PCM addition into cement-based concrete was investigated and reduced the compressive strength of the concrete up to a value of 30 MPa after 28 days, although it exhibited good thermal performance. Due to increased thermal inertia, the back-surface temperature of the sample with PCM was almost 5 °C lower compared to the reference during heating but the effect was detrimental in the cooling phase. The surface temperature of the PCM sample concrete was higher as compared to the reference in cooling because the PCM component will take more time to release the stored heat in the form of latent heat of fusion [23,24,25]. The thermal performance of a PCM-integrated gypsum wallboard was examined through a full-scale test room in Montreal, Canada. The PCM wallboard containing 25 wt % of paraffin showed a reduction in peak indoor temperature of 4 °C compared to the reference test room [26]. The performance enhancement of PCM wallboard was studied experimentally in a small test room for the application of passive solar buildings. The authors reported a reduction of 2 °C and 1.3 °C for inner surface temperature and indoor air temperature, respectively, for PCM-integrated wallboard compared to ordinary gypsum wallboard [27]. Other experimental studies on the thermal performance of PCM in concrete floors showed that concrete floors containing PCM reduce the maximum floor temperature by up to 16 ± 2% and increase the minimum temperatures by up to 7 ± 3% [28]. The thermal performance enhancement of PCM-integrated plastering mortars was investigated with the aid of experimental and numerical approaches. The pilot-scale experiment of this research showed a 2 °C reduction in the peak indoor temperature, while numerical studies assisted in optimizing the thermophysical properties of PCM-enhanced plastering mortars [29]. The thermal behavior of plastering mortars containing hybrid micro-encapsulated PCM was researched as a potential application to the interior surface of building walls. The study showed a maximum reduction of peak indoor temperature by 1.5 °C and increase in minimum temperature of 2.6 °C for the prototype test room experiments [30,31]. 

Many studies [21,28,32,33,34] have investigated the compatibility issue between PCMs and concrete mixture in order to minimize the effects of PCMs on macro thermomechanical performance after they are added into concrete mixture. Temperature peak of hydration can be reduced by up to 28.1% with the addition of 5% PCM capsules [21]. The combination of PCMs with concrete mixture can obtain an overall heat capacity 10 times higher than that of gypsum wall boards [35]. 

Geopolymer concrete (GPC) is an emerging construction material that can effectively handle industrial waste and reduce cementitious material, but its performance is least studied in terms of thermal enhancement. The addition of PCM into GPC has dropped the air temperature by 1.9 °C as compared to the reference [36]. PCM capsules were integrated into GPC and characterized for thermal and structural properties. Furthermore, the structure of the GPC was explained using SEM technique to link the microstructure with the structural properties [37,38]. It was reported that the thermal benefits of PCM addition were greater in the GPC as compared to OPC, but the authors experienced the problem of agglomeration due to PCM [37]. Another study reported that 20% addition of PCM still produced GPC, with sufficient compressive strength for most building components. At the same time, it reduced the weight of the building, hence generating a lightweight building. Additional benefits of PCM addition were enhanced heat capacity of GPC and reduction in heat transmission across the building component [39]. Polyester resin was used for the coating of PCM-impregnated expanded lightweight aggregate (ELA) and the capsules were integrated into GPC. The coating material increased the thermal conductivity of the capsules 42% higher as compared to the empty ELA. This increase in thermal conductivity is detrimental to the passive design of buildings [40]. A geopolymer mortar containing PCM in EP was produced and it was reported that peak temperature damping and reduction in variation of temperature profiles was observed [41]. A decrease in temperature of 6.8 °C and reduction in thermal conductivity with the addition of PCM into GPC had been achieved [42]. Paraffin-based PCM in EP was used as a replacement for fine aggregate in different ratios in ordinary cement mortar. With the 80% replacement, thermal energy storage rate and thermal energy storage capacity was increased by 56% and 166% as compared to pure cement mortar [43]. Cao et al. reported that the state of PCM inside the shell material had effects on the thermal and mechanical properties of the geopolymer concrete. The compressive strength of the specimens decreased with the phase transition of PCM from solid to liquid [44]. Furthermore, Cao et al. developed a model to quantify the effects of the PCM addition into concrete on the energy performance and simulated the climatic conditions of Madrid and Oslo. The study reported that wall orientation and seasonal behavior affect the energy performance of the buildings [45].

In our previous article [46], we aimed at the development of a leakproof geopolymer coating around the PCM for building applications. The integrity of the coating and its robustness in leak-proofing was tested and detailed in a previous published work [46]. Furthermore, the properties of the PCM are listed in Table A1 as claimed by the manufacturer. The current article deals with the effect of produced capsules on the thermal and structural performance of concrete embedded with the capsules. 

In the present study, geopolymer-coated capsules of PCM in foam, named GP-F-PCM, are incorporated into geopolymer concrete at the volume ratios of 25%, 50%, and 75% to develop thermally enhanced alkali-activated geopolymer concrete (GPC). Counterparts’ ratios are also prepared by using the same quantities of foam, only to compare the effects. The thermal and structural properties of the GPC are then investigated. The study produced a final element for building envelopes, presenting the raw materials and preparation methods for performance appraisal and final testing.

## 2. Materials and Methods

Alkali-activated geopolymer concrete (GPC) is developed using dune sand (DS) and the industrial waste of fly ash (FA) and granulated blast furnace slag (GGBS). Geopolymerization was activated using strong alkali solutions of sodium hydroxide and sodium silicate. The below sections provide details about the properties of the materials and the procedure.

### 2.1. Materials

**Figure 1:**
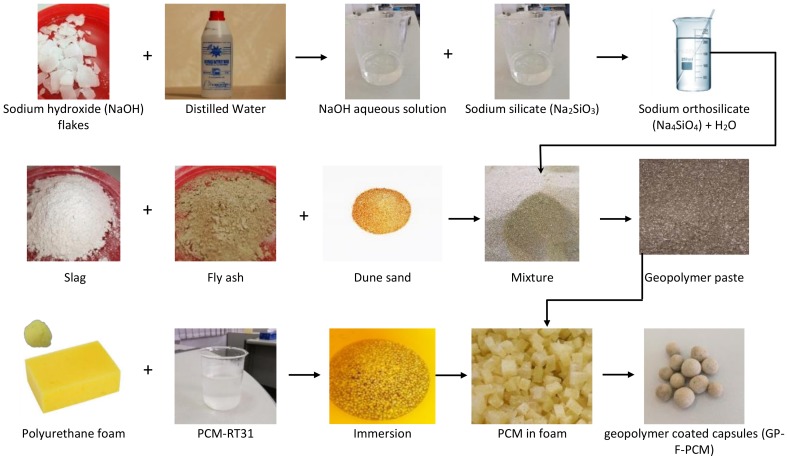
Photographs of the raw materials and the process of development of PCM capsules.

In the study, dune sand (DS) was used because it is an abundantly available material in the region of study (Al Ain, United Arab Emirates—24.21 °N, 55.74 °E). It had SiO_2_, CaO, and Al_2_O_3_ as major components with proportions of 63.9%, 14.1%, and 3%, respectively, while other oxides of iron, magnesium, sodium, and potassium were present as traces. FA was procured from Ashtech International (Dubai, United Arab Emirates). It had SiO_2_, Al_2_O_3_, Fe_2_O_3_, and CaO as major components, with amounts of 48%, 23.1%, 12.5%, and 3.3%, respectively. The remaining contents consisted of several components in minute quantities. Ground granulated blast furnace slag (GGBS) was from the Emirates Cement Factory (Al Ain, United Arab Emirates). Among the constituents of GGBS were CaO, SiO_2_, Al_2_O_3_, and MgO as prime contents, with quantities of 42%, 34.7%, 14.4%, and 6.9%, respectively. Densities of FA, GGBS and DS were 1262 kg/m^3^, 1236 kg/m^3^, and 1693 kg/m^3^, respectively, while their mean particle sizes were 2 µm, 12 µm, and 100 µm, respectively. Sodium silicate solution (Na_2_SiO_3_) and 97% pure flakes of sodium hydroxide (NaOH) were purchased from Sigma-Aldrich St. Louis, MO, USA. Phase change material (PCM) named as RT-31 was procured from Rubitherm, Berlin, Germany. The thermophysical properties of PCM are provided in Table A1. Polyurethane foam was used as a matrix material to hold PCM, which was eventually coated with geopolymer paste to yield form-stable PCM capsules named GP-F-PCM. The development procedure of the capsules and the materials characterization results (microstructure, composition, and physical properties) are detailed in [46] and [47]. All the raw materials used in the development of GP-F-PCM capsules and the process of capsule development are shown in Figure 1.

### 2.2. Specimen Preparation for Thermal and Structural Testing

**Figure 2:**
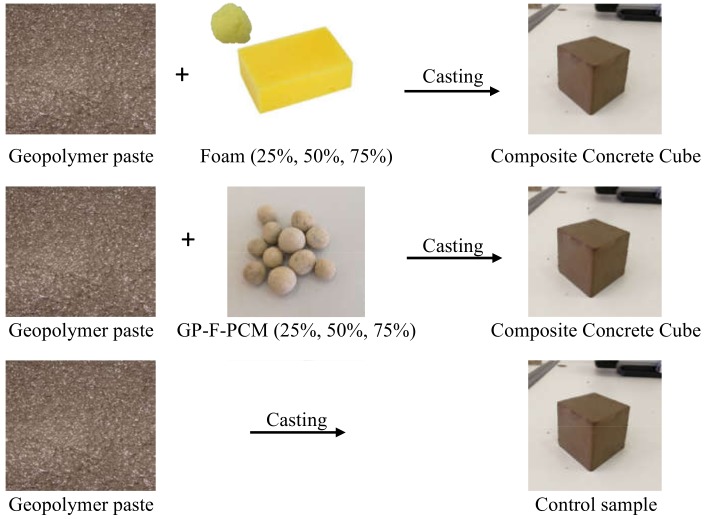
Representation of the development of concrete cubes.

Surface of the steel mold used for GPC casting was lubricated with oil for easy demolding. Cubes of GPC were cast with the dimensions of 50 mm × 50 mm × 50 mm. Foam and GP-F-PCM capsules were added to GPC cubes in the proportions of 25%, 50%, and 75%, as shown in Figure 2. In the case of additives (25%, 50%, and 75% for foam and GP-F-PCM), an equal volume of solid contents was removed. A control sample of pure geopolymer composition was developed to compare the thermal and structural performance against the composite concrete cubes. The ratios of the constituents of the coating are shown in Table 1.

### 2.3. Thermal Performance of GPC

**Figure 3:**
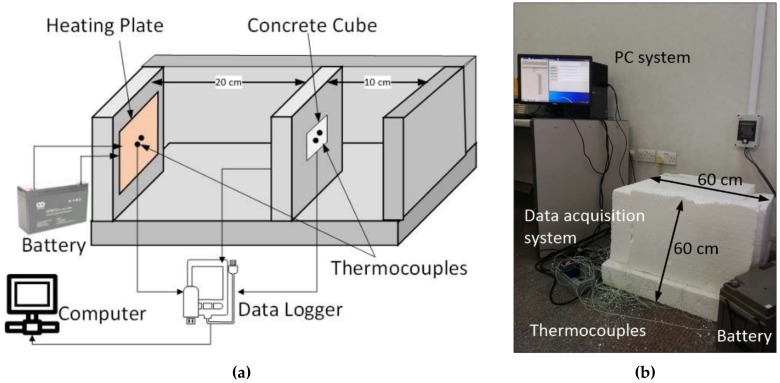
Experimental setup for thermal performance measurement: (**a**) schematic diagram and (**b**) original photo.

An enclosure 400 × 400 × 400 mm (as inner space) in size was designed to mimic an indoor setup employing 100 mm thick expanded polystyrene sheet. The polystyrene sheets were joined using insulating epoxy to prevent air infiltration from all but one side. The top side was kept operable to install measuring sensors and replace samples. An 80-Watt silicon heating mat, being 400 × 200 mm^2^, affixed to a 50 mm thick wooden sheet of the same dimensions, was used to supply heat to the setup. Samples were placed at calibrated distances of 220 mm from the heat source to represent certain heat loads. The heating setup was powered by a 12V, 150 Ah, rechargeable battery. A stable power output was maintained from the battery to assure consistent heat load on the sample during each set of experiments. Figure 3 shows a schematic diagram and photo of the experimental setup.

Temperatures were measured on all six sides of the test sample by installing two k-type thermocouples at the center of each side. The data were recorded with a time step of 1 min by a Compact DAQ (NI-cDAQ-9178, Austin, Texas, United States) data acquisition system interfaced with LabVIEW using analogue temperature module NI-9213. Experiments were conducted until steady-state temperature was achieved in both battery-powered heating and natural ambient cooling cycles. 

### 2.4. Thermal Transmittance (U-Value) Measurement

Thermal transmittance (U-value) of all different compositions was measured using a U-value kit from gSKIN^®^ green TEG, Zürich, Switzerland, as summarized in Table A2 [48]. The heat flux sensor was attached to rear side surface of the GPC cubes. Ambient temperature was measured 50 mm from both surfaces (front and rare) of the sample cubes. The U-value is calculated by employing Equation (1):

(1) $$U=\frac{\sum_{j=1}^{n}q_{j}}{\sum_{j=1}^{n}\left(T_{\mathrm{ij}}-T_{\mathrm{ej}}\right)},$$

where q_j_ = heat flux at time j, T_ij_ = inside air temperature at time j, T_ej_ = outside air temperature at time j.

### 2.5. Compressive Strength of Geopolymer Cubes

Compressive strength of all the specimens was tested using a 2000 kN Universal Testing Machine (Wykeham Farrance Engineering, Hertfordshire, UK) after seven and 28 days of curing. During preparation and curing, the specimens were kept at identical indoor conditions to minimize the variation of external effects. Strain endpoint and test point values were 0.8 mm/mm and 1.00 mm/min while applying load.

## 3. Results and Discussion

The behavior of developed GPC exposed to diurnal heat flux is the most important parameter of investigation. The results are supported by the U-value of the same composite geopolymer concrete cubes. Compressive strength is also presented to confirm which composition will conform with the building standards.

### 3.1. Thermal Performance

**Figure 4:**
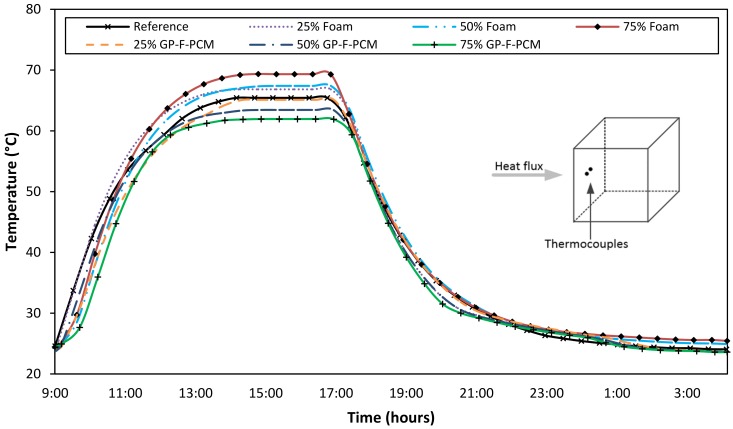
Front-surface temperature profiles.

Figure 4 represents the temperature profile for the front surface of GPC cubes for a complete heating and cooling cycle. At the start of the experiment, the cubes were at room temperature, which started rising at different rates when the heating was turned on. The rate of temperature rise is different for all the cubes because of the different material properties, but the overall trend is same. With the increase in the quantity of foam, the heat conduction of the cube is increased with respect to the reference cube. Steady-state temperature for the reference cube was achieved at 65.4 °C, while it was 66.9 °C, 67.4 °C, and 69.4 °C with the addition of 25%, 50%, and 75% of the foam, respectively. With the addition of GP-F-PCM, front-surface temperature decreased with the amount of increasing PCM. The surface temperature was 65.1 °C, 63.4 °C, and 61.9 °C for 25%, 50%, and 75% GP-F-PCM capsules, respectively. The difference in the front-surface temperatures is due to the back effect caused by the different thermal inertia of concrete composites.

**Figure 5:**
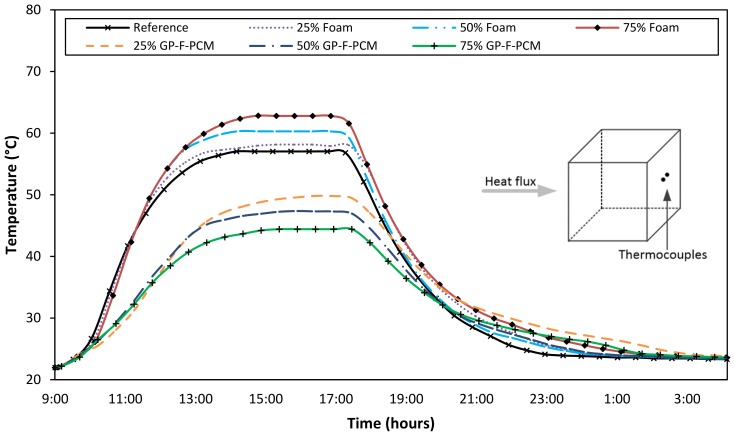
Back-surface temperature profiles.

The effect of different compositions on temperature drop is clearer in the back-surface temperature profiles. The overall trend of the temperature curves is like the front-surface temperature but with reduced magnitude. For the reference case, steady state was achieved at 57 °C, while it was 62.8 °C for 75% foam at one extreme and 44.4 °C at the other extreme for 75% GP-F-PCM capsules. Steady states for other composites lay between these two extremes, above and below the reference for foam and PCM, respectively, as shown in Figure 5. It is an unexpected finding that the addition of polyurethane foam in the mix of geopolymer concrete during homogenization increased the thermal transmittance of the produced cube as compared to the reference cube. However, the foam has very low thermal conductivity and is used for thermal insulations in buildings. In the literature, it is found that polyurethane foam reacts with NaOH and gets degraded into low molecular compounds [49,50]. This decomposition of foam may have caused the increase in surface temperature; however, further research is needed to establish the fact.

**Figure 6:**
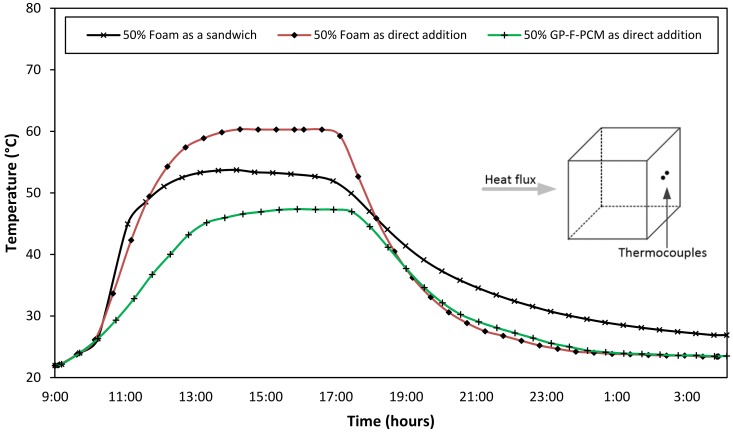
Back-surface temperature comparison of sandwich foam with the direct addition of foam and GP-F-PCM.

To validate the insulation effect of foam, a sandwich layer between two halves of GPC cube was developed with identical mass and total concrete thickness of 50 mm. A 50% volume of foam based on previous composition was compressed tightly in between two halves of pure GPC and exposed to the same heat flux. Figure 6 shows the comparison of back-surface temperatures of the sandwich cube with the direct addition of foam and GP-F-PCM capsules. The back-surface temperature for sandwich cube is 53.7 °C and that of 50% foam inside the composition is 61 °C. It can be concluded that foam has insulation effect if it is used as a sandwich layer, but it has an adverse effect on heat transmission if incorporated into GPC because of the reaction with alkali.

**Figure 7:**
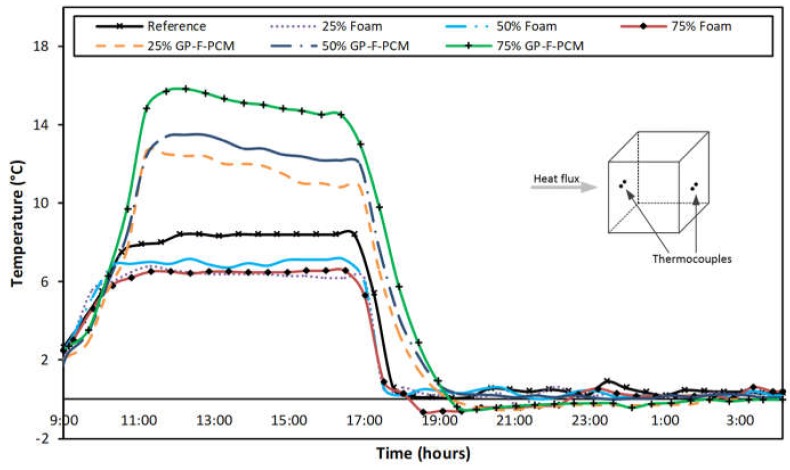
Differences in front-surface and back-surface temperature.

One important benefit obtained by using PCM in building components is the delay in heat progression into concrete employing PCM. To illustrate it in the current research, the difference of temperatures on the front and back surface for all the samples is plotted in Figure 7 on the same time scale. From 10:00 to 11:00, the prevention of heat propagation in the samples with 75% capsules is approximately 9 °C as compared to the reference sample. This means that the temperature gradient is low in cubes containing PCM samples, which is why the difference between front and back is higher as compared to all other samples. The same is the case for other samples containing PCM amounts of 50% and 25% but with lower magnitude. After closing the heating cycle and allowing the samples to cool through natural convection, the temperature stayed higher for a longer time for the PCM cases because of the higher thermal inertia in the time range of 17:00 to 19:00.

**Figure 8:**
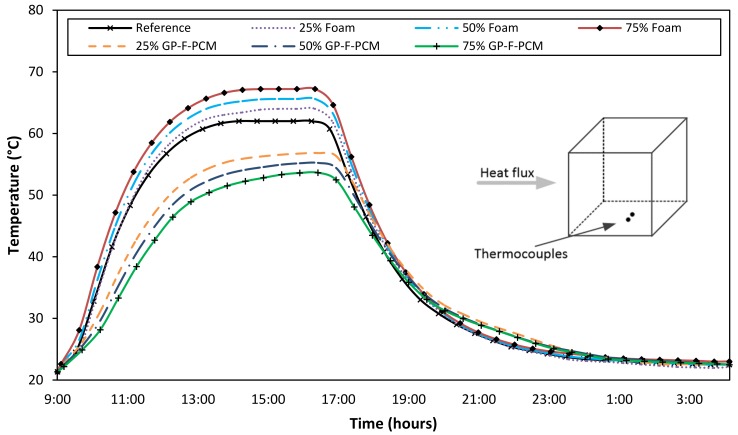
Comparison of temperature variations at one side of all the cubes.

Figure 8 shows the temperature profile obtained from the sensors attached to one side of the cubes. Temperature behavior is again like the front and the back surface, but the values of these are in between the two. Steady state of the temperature curves was achieved at 67.2 °C, 62.0 °C, and 53.6 °C for the 75% foam, reference, and 75% GP-F-PCM capsules, respectively. 

**Figure A1:**
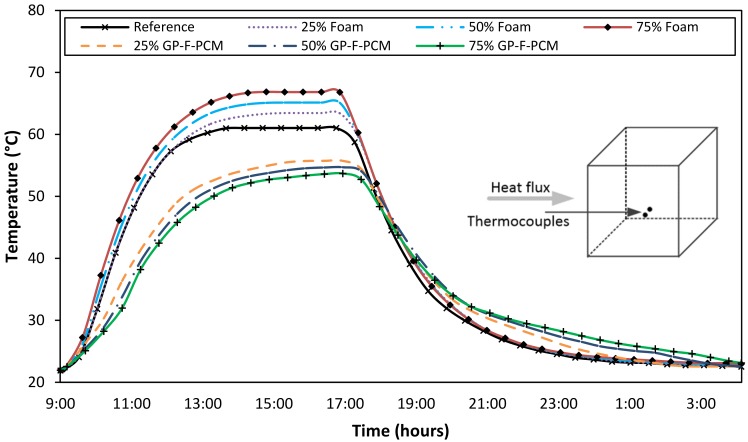
Comparison of temperature variations at the 2nd side of all the cubes.

**Figure A2:**
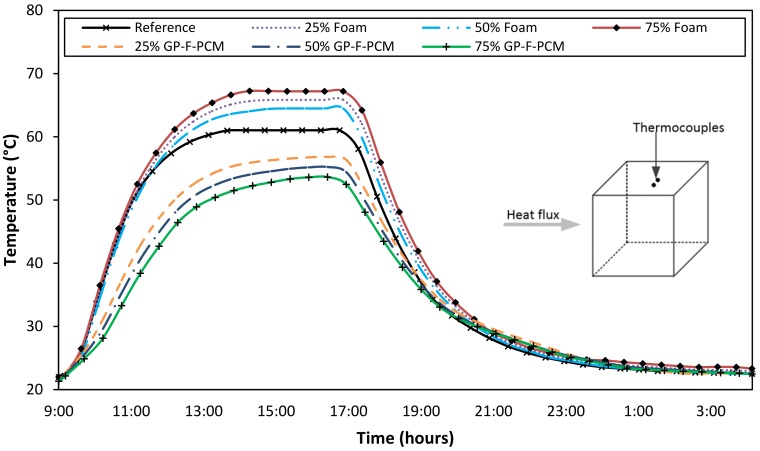
Comparison of temperature variations at the 3rd side of all the cubes.

**Figure A3:**
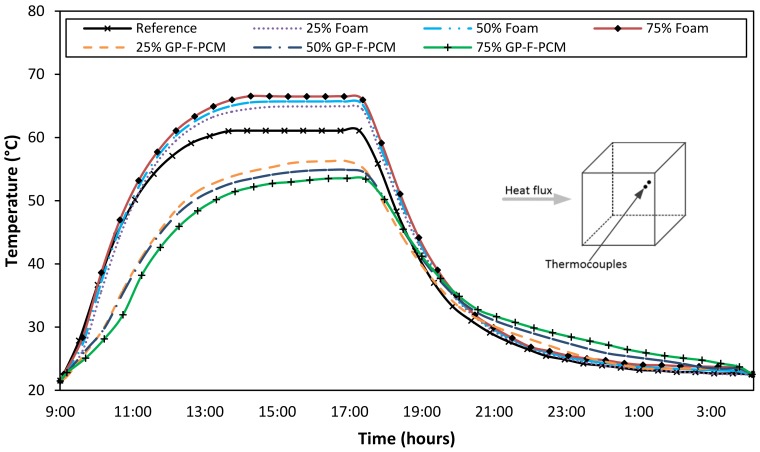
Comparison of temperature variations at the 4th side of all the cubes.

For the other three sides, temperature behavior was also same, and the corresponding figures are shown in Figure A1, Figure A2 and Figure A3, while steady-state temperatures at all surfaces and for all composite concrete cubes are summarized in Table 2. A high precision among temperature values is due to the uniform distribution of foam/GP-F-PCM capsules. This uniformity was also confirmed by cutting a slice, as represented in the structural part of the thesis [47]. 

### 3.2. Thermal Transmittance (U-Value)

**Figure 9:**
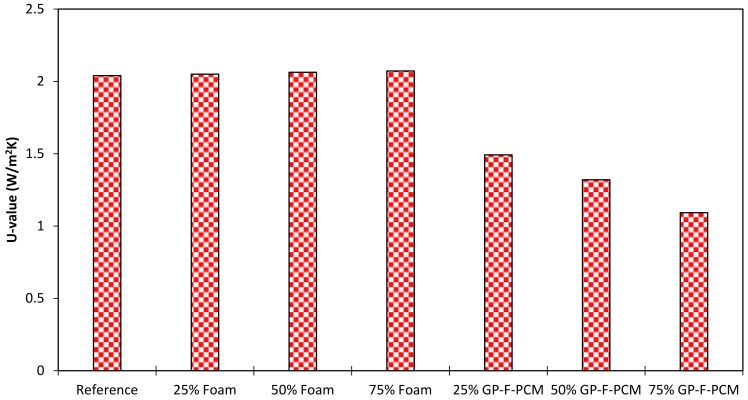
U-value of GPC blocks measured experimentally.

Figure 9 represents the results of U-value for all geopolymer concrete compositions. The software provided by green TEG integrates the measured data over the entire time domain and gives a single U-value based on Equation (1). The highest value of 2.07 W/m^2^K is observed for 75% foam (even greater than the reference value of 2.04 W/m^2^K), which may be attributed to increased thermal conductivity due to the reaction of the foam with the alkali. Another reason could be the increase in density of the geopolymer reaction products, because it also increased the structural strength, as described in the compressive results section. With the inclusion of GP-F-PCM, the insulation effect of the material is improved. In comparison to the reference, U-values decreased by 27%, 35%, and 47% with the addition of GP-F-PCM in proportions of 25%, 50%, and 75%, respectively.

### 3.3. Compressive Strength 

**Figure 10:**
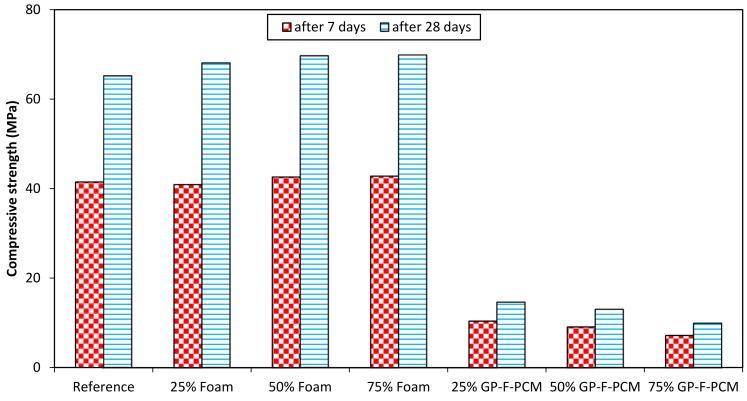
Compressive strength test results.

Figure 10 shows the compressive strength of GPC after seven and 28 days with different amounts of foam and GP-F-PCM. The compressive strength was 41.5 MPa and 65.7 MPa for the reference case after seven and 28 days, respectively. The strength was like the reference cube in the case of addition of foam, where it ranged from 40.9 MPa to 42.8 MPa at the age of seven days and 68.1 MPa to 69.9 MPa at the age of 28 days. Compressive strength dropped with the inclusion of GP-F-PCM capsules, which ranged from 7.2 MPa to 10.4 MPa after seven days and 9.9 MPa to 14.6 MPa after 28 days. Comparing the composite concrete of foam with the reference cube, specimens with foam exhibited slightly more strength, which may be attributed to more compaction. In the case of GP-F-PCM, more fragile areas are developed due to the presence of PCM capsules [40]. Cui et al. pointed out the low shear strength and stiffness of PCM capsules responsible for the loss of compressive strength [42]. Results of the previous studies reported that the addition of PCM capsules induced porosity into GPC [37]. This porosity is linked with the reduction in compressive strength [37]. Other researchers stated that PCM adversely affected the geopolymerization process of FA, which caused a reduction in compressive strength [39].

The impact of curing time from seven days to 28 days is increased compressive strength of 63.3% in the current study, while there are different findings reported in the literature. Gain in strength at the age of 28 days as compared to seven days is 14% higher [39], more than 1.5 times [51], 51.4% higher [52], and almost no change [53], while a few of the studies only investigated after seven days as they considered that most of the strength can be achieved after aging for seven days [54,55]. The international standard, ASTM C129-17 [56], requires nonloadbearing concrete units to achieve a compressive strength of 4.14 MPa. The developed GP-F-PCM exhibits a compressive strength suitable for nonloadbearing walls and claddings.

## 4. Conclusions

In the present study, phase change material capsules are produced by the immersion of PCM into polyurethane foam and coated with geopolymer. Stability of the capsules was tested by rapid thermal cycling and weathering tests. The effect of addition of polyurethane foam and geopolymer coated PCM capsules of foam (GP-F-PCM) on the thermal and structural performance of geopolymer concrete (GPC) was investigated experimentally. Foam and GP-F-PCM were added in 25%, 50%, and 75% proportions by volume. The results of the prepared composite cubes were compared with the reference/control GPC cube. The addition of foam into GPC increased the back-surface temperatures of the cubes and the increasing trend was observed by increasing the content of the foam. For the maximum amount of foam (75%), back-surface temperature increased by 5.9 °C in comparison to the reference concrete. Addition of GP-F-PCM to the GPC dropped the back-surface temperatures and a further temperature drop was achieved by increasing the PCM content. A temperature drop of 12.4 °C was measured at the back surface for 75% GP-F-PCM concrete compared to the reference. This research revealed that polyurethane foam as a sandwich layer or GP-F-PCM can reduce heat transmissions indoor but direct integration of foam into GPC is detrimental. U-value of the composite concretes was also measured experimentally to validate the results of thermal performance. Addition of 75% foam has increased the U-value by 2.5%; however, the addition of 75% of GP-F-PCM capsules decreased it by 47%. Incorporation of 75% foam slightly increased the strength of GPC cube (+3.6%) as compared to the reference cube, while it was significantly reduced when PCM capsules were incorporated. For 28 days curing time, the compressive strength dropped from 65.2 MPa for the reference specimen to 9.9 MPa for 75% GP-F-PCM composites. This is attributed to the presence of fragile PCM inside the capsules. The results show that the incorporation of GP-F-PCM capsules achieved the best thermal performance when compared with a foam as a sandwich layer or the direct addition of foam. The characterization of thermophysical properties of the foam-PCM capsules is further research that is underway and will form the basis for theoretical investigations. 

## Figures and Tables

**Table 1:** Geopolymer composition (mass ratio, kg/m^3^).

Fly Ash	Slag	Dune Sand	NaOH (18M Solution)	Na_2_SiO_3_	Total
457	152	914	101	252	1876

**Table 2:** Steady-state temperatures at different surfaces for all the geopolymer concrete composites.

	Reference (°C)	25% Foam (°C)	50% Foam (°C)	75% Foam (°C)	25% GP-F-PCM (°C)	50% GP-F-PCM (°C)	75% GP-F-PCM (°C)
Front surface	65.4	66.9	67.4	69.4	65.1	63.4	61.9
Back surface	57	58.2	60.4	62.9	49.9	47.4	44.5
Back surface for sandwich	NA	NA	53.7	NA	NA	NA	NA
Side 1	62	64	65.6	67.2	56.8	55.2	53.7
Side 2	61.1	63.4	65.2	66.9	55.7	54.7	53.8
Side 3	61.1	64.5	65.8	67.3	56.8	55.2	53.7
Side 4	61.2	64.9	65.7	66.6	56.2	54.9	53.5

## Nomenclature

ELA	expanded lightweight aggregate
EP	expanded perlite
FA	fly ash
GGBS	ground granulated blast-furnace slag
GPC	geopolymer concrete
GP-F-PCM	geopolymer-coated, form-stable capsules of PCM in foam
NaOH	sodium hydroxide
Na_2_SiO_3_	sodium silicate
Na_4_SiO_4_	sodium ortho silicate
LTES	latent thermal energy storage
OPC	ordinary Portland cement
PCM	phase change material

## Appendix A

**Table A1:** Properties of the PCM-RT31 [57].

Parameters	Typical Values
Melting area	27–33 [°C]
Congealing area	33–27 [°C]
Heat storage capacity ± 7.5%	165 [kJ/kg]
Specific heat capacity	2 [kJ/kg.K]
Density solid	0.88 [kg/L]
Density liquid	0.76 [kg/L]
Heat conductivity (both phases)	0.2 [W/m.K]
Volume Expansion	12.5%
Flash point	157 [°C]

**Table A2:** Features of U-value kit used in the experiment [48].

Description of Parameter	Value
Product Name	gSKIN^®^ KIT-2615C
Heat Flux Range Min/Max [W/m^2^]	±200
Heat Flux Resolution [W/m^2^]	<0.22
Min. Sensor Sensitivity (S) [µV/(W/m^2^)]	7
Temperature Sensor Accuracy [°C]	±0.5 (−10…+65 °C) ±2.0 (−55…+125 °C)
Measurement Frequency	1/s to 1/h
Operating Temperature Range Min/Max [°C]	−40/100 (−20/65 for Logger)
Calibration Temperature Range Min/Max [°C]	−30/70
Calibration Accuracy [±%]	3

## References

[1] Zhu N., Ma Z., Wang S. (2009). Dynamic characteristics and energy performance of buildings using phase change materials: A review. Energy Convers. Manag..

[2] Patterson R. World Energy 2016–2050: Annual Report. http://content.csbs.utah.edu/~mli/Economies%205430-6430/World%20Energy%202016-2050.pdf.

[3] Ürge-Vorsatz D., Cabeza L.F., Serrano S., Barreneche C., Petrichenko K. (2015). Heating and cooling energy trends and drivers in buildings. Renew. Sustain. Energy Rev..

[4] Guichard S., Miranville F., Bigot D., Malet-Damour B., Beddiar K., Boyer H. (2017). A complex roof incorporating phase change material for improving thermal comfort in a dedicated test cell. Renew. Energy.

[5] Rashid Y., Alnaimat F., Mathew B. (2018). Energy Performance Assessment of Waste Materials for Buildings in Extreme Cold and Hot Conditions. Energies.

[6] Zhang B., Sun Q.H., Liu W.T. (2016). Thermal Performance of a Shape-Stabilized Phase Change Material Floor with Different Heating Positions. Key Eng. Mater..

[7] Cellat K., Kazanci B., Konuklu Y., Paksoy H. (2017). Direct Incorporation of Butyl Stearate as Phase Change Material into Concrete for Energy Saving in Buildings. J. Clean Energy Technol..

[8] Hassan A., Shakeel Laghari M., Rashid Y. (2016). Micro-Encapsulated Phase Change Materials: A Review of Encapsulation, Safety and Thermal Characteristics. Sustainability.

[9] Soares N., Costa J.J., Gaspar A.R., Santos P. (2013). Review of passive PCM latent heat thermal energy storage systems towards buildings’ energy efficiency. Energy Build..

[10] Medina M., King J., Zhang M. (2008). On the heat transfer rate reduction of structural insulated panels (SIPs) outfitted with phase change materials (PCMs). Energy.

[11] Ahmad M., Bontemps A., Sallée H., Quenard D. (2006). Thermal testing and numerical simulation of a prototype cell using light wallboards coupling vacuum isolation panels and phase change material. Energy Build..

[12] Zwanzig S.D., Lian Y., Brehob E.G. (2013). Numerical simulation of phase change material composite wallboard in a multi-layered building envelope. Energy Convers. Manag..

[13] Ghedamsi R., Settou N., Saifi N., Dokkar B. (2014). Contribution on Buildings Design with Low Consumption of Energy Incorporated PCMs. Energy Procedia.

[14] Hasan A., Al-Sallal K., Alnoman H., Rashid Y., Abdelbaqi S. (2016). Effect of Phase Change Materials (PCMs) Integrated into a Concrete Block on Heat Gain Prevention in a Hot Climate. Sustainability.

[15] Silva T., Vicente R., Soares N., Ferreira V. (2012). Experimental testing and numerical modelling of masonry wall solution with PCM incorporation: A passive construction solution. Energy Build..

[16] Bontemps A., Ahmad M., Johannès K., Sallée H. (2011). Experimental and modelling study of twin cells with latent heat storage walls. Energy Build..

[17] Kara Y.A., Kurnuç A. (2012). Performance of coupled novel triple glass unit and pcm wall. Appl. Therm. Eng..

[18] Silva S.M., Manuela G.A., Bragança L. (2011). Evaluation of the Thermal Performance of Hollow Brick Walls with Gypsum-PCM Plasters.

[19] Li X., Sanjayan J.G., Wilson J.L. (2014). Fabrication and stability of form-stable diatomite/paraffin phase change material composites. Energy Build..

[20] Li H., Chen H., Li X., Sanjayan J.G. (2014). Development of thermal energy storage composites and prevention of PCM leakage. Appl. Energy.

[21] Hunger M., Entrop A.G., Mandilaras I., Brouwers H.J.H., Founti M. (2009). The behavior of self-compacting concrete containing micro-encapsulated Phase Change Materials. Cem. Concr. Compos..

[22] Ramakrishnan S., Sanjayan J., Wang X., Alam M., Wilson J. (2015). A novel paraffin/expanded perlite composite phase change material for prevention of PCM leakage in cementitious composites. Appl. Energy.

[23] Xu B., Li Z. (2014). Performance of novel thermal energy storage engineered cementitious composites incorporating a paraffin/diatomite composite phase change material. Appl. Energy.

[24] Xu B., Li Z. (2013). Paraffin/diatomite composite phase change material incorporated cement-based composite for thermal energy storage. Appl. Energy.

[25] Hasan A., Alnoman H., Rashid Y. (2016). Impact of integrated photovoltaic-phase change material system on building energy efficiency in hot climate. Energy Build..

[26] Athienitis A.K., Liu C., Hawes D., Banu D., Feldman D. (1997). Investigation of the thermal performance of a passive solar test-room with wall latent heat storage. Build. Environ..

[27] Sarı A., Karaipekli A., Kaygusuz K. (2008). Capric acid and stearic acid mixture impregnated with gypsum wallboard for low-temperature latent heat thermal energy storage. Int. J. Energy Res..

[28] Entrop A.G., Brouwers H.J.H., Reinders A.H.M.E. (2011). Experimental research on the use of micro-encapsulated Phase Change Materials to store solar energy in concrete floors and to save energy in Dutch houses. Sol. Energy.

[29] Sá A.V., Azenha M., de Sousa H., Samagaio A. (2012). Thermal enhancement of plastering mortars with Phase Change Materials: Experimental and numerical approach. Energy Build..

[30] Kheradmand M., Azenha M., de Aguiar J.L.B., Krakowiak K.J. (2014). Thermal behavior of cement based plastering mortar containing hybrid microencapsulated phase change materials. Energy Build..

[31] Kheradmand M., Azenha M., de Aguiar J.L.B., Castro-Gomes J. (2016). Experimental and numerical studies of hybrid PCM embedded in plastering mortar for enhanced thermal behaviour of buildings. Energy.

[32] Jeong S.-G., Jeon J., Cha J., Kim J., Kim S. (2013). Preparation and evaluation of thermal enhanced silica fume by incorporating organic PCM, for application to concrete. Energy Build..

[33] Memon S.A., Cui H., Lo T.Y., Li Q. (2015). Development of structural–functional integrated concrete with macro-encapsulated PCM for thermal energy storage. Appl. Energy.

[34] Navarro L., de Gracia A., Castell A., Álvarez S., Cabeza L.F. (2015). PCM incorporation in a concrete core slab as a thermal storage and supply system: Proof of concept. Energy Build..

[35] Baetens R., Jelle B.P., Gustavsen A. (2010). Phase change materials for building applications: A state-of-the-art review. Energy Build..

[36] Suttaphakdee P., Dulsang N., Lorwanishpaisarn N., Kasemsiri P., Posi P., Chindaprasirt P. (2016). Optimizing mix proportion and properties of lightweight concrete incorporated phase change material paraffin/recycled concrete block composite. Constr. Build. Mater..

[37] Cao V.D., Pilehvar S., Salas-Bringas C., Szczotok A.M., Rodriguez J.F., Carmona M., Al-Manasir N., Kjøniksen A.-L. (2017). Microencapsulated phase change materials for enhancing the thermal performance of Portland cement concrete and geopolymer concrete for passive building applications. Energy Convers. Manag..

[38] Pilehvar S., Cao V.D., Szczotok A.M., Valentini L., Salvioni D., Magistri M., Pamies R., Kjøniksen A.-L. (2017). Mechanical properties and microscale changes of geopolymer concrete and Portland cement concrete containing micro-encapsulated phase change materials. Cem. Concr. Res..

[39] Shadnia R., Zhang L., Li P. (2015). Experimental study of geopolymer mortar with incorporated PCM. Constr. Build. Mater..

[40] Kastiukas G., Zhou X., Castro-Gomes J. (2016). Development and optimisation of phase change material-impregnated lightweight aggregates for geopolymer composites made from aluminosilicate rich mud and milled glass powder. Constr. Build. Mater..

[41] Wang Z., Su H., Zhao S., Zhao N. (2016). Influence of phase change material on mechanical and thermal properties of clay geopolymer mortar. Constr. Build. Mater..

[42] Cui H., Feng T., Yang H., Bao X., Tang W., Fu J. (2018). Experimental study of carbon fiber reinforced alkali-activated slag composites with micro-encapsulated PCM for energy storage. Constr. Build. Mater..

[43] Ramakrishnan S., Wang X., Sanjayan J., Wilson J. (2017). Thermal energy storage enhancement of lightweight cement mortars with the application of phase change materials. Procedia Eng..

[44] Cao V.D., Pilehvar S., Salas-Bringas C., Szczotok A.M., Valentini L., Carmona M., Rodriguez J.F., Kjøniksen A.L. (2018). Influence of microcapsule size and shell polarity on thermal and mechanical properties of thermoregulating geopolymer concrete for passive building applications. Energy Convers. Manag..

[45] Cao V.D., Pilehvar S., Salas-Bringas C., Szczotok A.M., Bui T.Q., Carmona M., Rodriguez J.F., Kjøniksen A.L. (2019). Thermal analysis of geopolymer concrete walls containing microencapsulated phase change materials for building applications. Sol. Energy.

[46] Hassan A., Ismail N., Mourad A.-H., Rashid Y., Laghari M. (2018). Preparation and Characterization of Expanded Clay-Paraffin Wax-Geo-Polymer Composite Material. Materials.

[47] Rashid Y. (2018). Thermal and Structural Characterization of Macro-Encapsulated Phase Change Material Integrated into Concrete Cubes. Master’s Theses.

[48] https://www.greenteg.com/template/userfiles/files/gSKIN_KIT_U-Value_Datasheet_v3.6.pdf.

[49] Chuayjuljit S., Norakankorn C., Pimpan V. (2002). Chemical Recycling of Rigid Polyurethane Foam Scrap via Base Catalyzed Aminolysis. J. Met. Mater. Miner..

[50] Ismail E.A., Motawie A.M., Sadek E.M. (2011). Synthesis and characterization of polyurethane coatings based on soybean oil–polyester polyols. Egypt. J. Pet..

[51] Wallah S., Rangan B.V. (2006). Low-Calcium Fly Ash-Based Geopolymer Concrete: Long-Term Properties.

[52] Ismail N., Mansour M., El-Hassan H. (2017). Development of a low-cost cement free polymer concrete using industrial by-products and dune sand. MATEC Web Conf..

[53] Zhang L., Ahmari S., Zhang J. (2011). Synthesis and characterization of fly ash modified mine tailings-based geopolymers. Constr. Build. Mater..

[54] Ahmari S., Zhang L. (2012). Production of eco-friendly bricks from copper mine tailings through geopolymerization. Constr. Build. Mater..

[55] Ahmari S., Zhang L., Zhang J. (2012). Effects of activator type/concentration and curing temperature on alkali-activated binder based on copper mine tailings. J. Mater. Sci..

[56] (2017). ASTM C129-17, Standard Specification for Nonloadbearing Concrete Masonry Units.

[57] Technical Data Sheet of PCM-RT31. https://www.rubitherm.eu/media/products/datasheets/Techdata_-RT31_EN_31052016.PDF.

